# Sensory impairment and all-cause mortality among the elderly adults in China: a population-based cohort study

**DOI:** 10.18632/aging.202198

**Published:** 2020-11-26

**Authors:** Ji Sun, Lin Li, Jiangwei Sun

**Affiliations:** 1 Department of Pathology, Eye and ENT Hospital, Fudan University, Shanghai, China; 2 School of Medical Sciences, Örebro University, Örebro, Sweden; 3 Department of Medical Epidemiology and Biostatistics, Karolinska Institutet, Stockholm, Sweden; 4 Institute of Environmental Medicine, Karolinska Institutet, Stockholm, Sweden

**Keywords:** sensory impairment, mortality, cohort, elderly population, China

## Abstract

With age-related functional deterioration, sensory impairment including vision impairment (VI), hearing impairment (HI), and dual sensory impairment (DSI) usually occurred among the elderly population, causing a decrease in functional capacity and quality of life. The study aimed to explore how sensory impairment is associated with the risk of all-cause mortality among the elderly adults in China. We prospectively investigated the association among 37,076 participants enrolled from 1998 to 2019 in the Chinese Longitudinal Healthy Longevity Survey. We also, as a sensitivity analysis, explored the association among 11,365 newly incident sensory impairment participants. Cox regression model with sensory impairment as a time-varying exposure was performed to calculate the hazard ratios (HRs) and 95% confidence intervals (CIs). Compared with participants without sensory impairment, those with VI (HR=1.20, 95% CI: 1.15-1.24), HI (HR=1.26, 95% CI: 1.21-1.31), and DSI (HR: 1.46, 95% CI=1.41-1.52) had significant higher risk of all-cause mortality after adjusting for potential confounders. These associations were robust among subgroup analyses stratified by sex and entry age, and sensitivity analyses performed among newly incident sensory impairment participants. In conclusion, sensory impairment was associated with higher mortality risk among the elderly adults in China.

## INTRODUCTION

Most countries have been facing with the challenges of population aging due to the technological and medical development [1]. The proportion of population aged more than 65 years will rise from 12.4% in 2000 to 17.9% in 2050 in America, and from 13% in 2006 to 26% in 2050 in Australia [1, 2]. China is undoubtedly no exception. As reported, the elderly population in China will exceed 450 million in 2050, comprising more than 30% of the total population and nearly 25% of the world elderly population [3].

Although people are living longer and in better health at older ages than previously, the health status of the elderly people inevitable deteriorates and an increased number of the elderly people survives with more health problems, e.g., frailty, disability and noncommunicable diseases, which has been known as “failure or cost of success” [4]. As a type of age-related function loss, sensory impairment including vision impairment (VI), hearing impairment (HI), and dual sensory impairment (DSI) affect around 10% of the elderly population [5, 6], leading to partially or fully disconnect with surroundings [7, 8] and decreasing quality of life [9]. Previous studies have shown sensory impairment was associated with a variety of functional or clinical outcomes, including hip fracture, neurodegeneration, and cognitive impairment [5, 10–12].

The relationship between sensory impairment and all-cause mortality has also been explored in previous studies but with inconsistent results [13–16]. Besides, these studies were mainly focus on relatively younger (usually less than 80 years old) population, therefore, the aforementioned associations were still unclear among the oldest-old population including octogenarians (person with 80-89 years old), nonagenarians (person with 90-99 years old), or centenarians (person with more than 100 years old). Furthermore, previous studies usually only used the baseline sensory status to explore its influence on mortality risk, without accounting for the change of exposure status after enrollment, which could lead to misclassification of exposure status and inevitably cause bias [6].

Therefore, to tackle with the abovementioned knowledge gap, we hereby, using data from the Chinese Longitudinal Healthy Longevity Survey (CLHLS), investigated the association between sensory impairment and all-cause mortality among the elderly adults.

## RESULTS

The baseline characteristics of participants stratified by sensory impairment was shown in Table 1. Compared with participants without sensory impairment, participants with sensory impairment (HI, VI or DSI) tend to be older, more female, living in rural areas and town, and illiteracy. Those participants also have a higher percentage of ADL disability, physical performance disability, cognitive impairment, and social activity disengagement, but lower chronic disease score.

**Table 1:** Baseline characteristics of the elderly adults, a cohort study in China, 1998-2019.

**Variables**	**Sensory impairment**				
	**No sensory impairment (n=18,884)**	**VI only (n=5,141)**	**HI only (n=5,277)**	**DSI (n=7,774)**	**P value**
Age at baseline, years					<0.0001
Mean ± SD	83.29 ± 10.92	90.23 ± 10.35	94.03 ± 7.66	97.35 ± 6.55	
Median (IQR)	83.45 (74.61-91.25)	91.33 (83.83-100.00)	94.58 (89.19-100.60)	100.14 (93.09-101.65)	
Categories, n(%)					<0.0001
<80 years	6583 (34.86)	800 (15.56)	198 (3.75)	127 (1.63)	
80-89 years	6646 (35.19)	1416 (27.54)	1270 (24.07)	910 (11.71)	
90-99 years	3936 (20.84)	1638 (31.86)	2095 (39.70)	2644 (34.01)	
≥100 years	1719 (9.10)	1287 (25.03)	1714 (32.48)	4093 (52.65)	
Sex, n(%)					<0.0001
Male	9416 (49.86)	1797 (34.95)	2174 (41.20)	1962 (25.24)	
Female	9468 (50.14)	3344 (65.05)	3103 (58.80)	5812 (74.76)	
Residence, n(%)					<0.0001
City	4834 (25.60)	1027 (19.98)	1256 (23.80)	1451 (18.66)	
Town	5429 (28.75)	1510 (29.37)	1752 (33.20)	2336 (30.05)	
Rural area	8621 (45.65)	2604 (50.65)	2269 (43.00)	3987 (51.29)	
Ethic, n(%)*					<0.0001
Han	17465 (94.13)	4714 (92.98)	4999 (96.01)	7365 (95.91)	
Others	1089 (5.87)	356 (7.02)	208 (3.99)	314 (4.09)	
Marriage status, n(%)*					<0.0001
Married	11198 (59.33)	3975 (77.38)	4419 (83.77)	7090 (91.22)	
Others	7677 (40.67)	1162 (22.62)	856 (16.23)	682 (8.78)	
Occupation, n(%)*					<0.0001
Farmer or manual	10122 (53.85)	3151 (61.52)	2745 (52.25)	4773 (61.75)	
Clerical	5111 (27.19)	1225 (23.92)	1614 (30.72)	1885 (24.39)	
Professional	1748 (9.30)	180 (3.51)	220 (4.19)	161 (2.08)	
Others	1814 (9.65)	566 (11.05)	675 (12.85)	911 (11.79)	
Education, n(%)*					<0.0001
Illiterate	10252 (54.51)	3706 (72.65)	3811 (72.56)	6474 (84.22)	
Primary school	6236 (33.16)	1133 (22.21)	1145 (21.80)	1015 (13.20)	
Middle school or above	2319 (12.33)	262 (5.14)	296 (5.64)	198 (2.58)	
Access to medical service, n(%)*					<0.0001
Yes	16375 (86.77)	4343 (84.51)	4495 (85.21)	6472 (83.27)	
No	2497 (13.23)	796 (15.49)	780 (14.79)	1300 (16.73)	
Smoking status, n(%)*					<0.0001
Never	11711 (62.10)	3722 (72.48)	3583 (68.05)	6128 (78.98)	
Ever smoker	2787 (14.78)	641 (12.48)	799 (15.18)	854 (11.01)	
Current smoker	4361 (23.12)	772 (15.03)	883 (16.77)	777 (10.01)	
Drinking status, n(%)*					<0.0001
Never	12459 (66.10)	3722 (72.50)	3537 (67.17)	5868 (75.69)	
Ever drinker	1936 (10.27)	496 (9.66)	548 (10.41)	747 (9.63)	
Current drinker	4453 (23.63)	916 (17.84)	1181 (22.43)	1138 (14.68)	
Exercise status, n(%)*					<0.0001
Never	11258 (59.76)	3602 (70.27)	3615 (68.77)	6009 (77.58)	
Ever exerciser	974 (5.17)	392 (7.65)	418 (7.95)	795 (10.26)	
Current exerciser	6606 (35.07)	1132 (22.08)	1224 (23.28)	942 (12.16)	
ADL score, n(%)*					<0.0001
6	16418 (87.18)	3543 (69.06)	3352 (63.91)	3162 (40.81)	
5	1423 (7.56)	684 (13.33)	905 (17.25)	1180 (15.23)	
3-4	550 (2.92)	447 (8.71)	499 (9.51)	1150 (14.84)	
0-2	441 (2.34)	456 (8.89)	489 (9.32)	2256 (29.12)	
Physical performance score, n(%)*					<0.0001
5	13213 (70.24)	2046 (40.11)	2065 (39.31)	1084 (14.10)	
2.5-4.5	5194 (27.61)	2554 (50.07)	2761 (52.56)	4314 (56.13)	
0-2.5	405 (2.15)	501 (9.82)	427 (8.13)	2288 (29.77)	
MMSE score, n(%)*					<0.0001
24-30	13768 (78.11)	2288 (47.27)	1589 (32.18)	732 (10.10)	
18-23	2819 (15.99)	1394 (28.80)	1167 (23.63)	1034 (14.27)	
0-17	1040 (5.90)	1158 (23.93)	2182 (44.19)	5482 (75.63)	
Food diversity score, n(%)*					<0.0001
6-8	9344 (50.12)	2058 (40.43)	2155 (41.59)	2550 (33.39)	
4-5	6352 (34.07)	1878 (36.90)	1961 (37.85)	2981 (39.03)	
0-3	2946 (15.80)	1154 (22.67)	1065 (20.56)	2106 (27.58)	
Social activity score, n(%)*					<0.0001
5-8	4861 (25.76)	532 (10.35)	1055 (20.01)	668 (8.60)	
3-4	9701 (51.41)	2356 (45.84)	2136 (40.51)	2463 (31.71)	
0-2	4307 (22.83)	2252 (43.81)	2082 (39.48)	4637 (59.69)	
Chronic disease score, n(%)*					<0.0001
0	10681 (58.60)	2968 (60.02)	3272 (64.23)	4858 (64.61)	
1-2	5875 (32.23)	1523 (30.80)	1481 (29.07)	2164 (28.78)	
≥3	1671 (9.17)	454 (9.18)	341 (6.69)	497 (6.61)	

Abbreviations: ADL: activities of daily living; DSI: dual sensory impairment; HI: hearing impairment; IQR, interquartile range; MMSE: Mini-Mental State Examination; SD standard deviation; VI: vision impairment.

*: contains missing values. Ethic: 566, marriage status:17, occupation: 175, access to medical service: 18, education:229, smoking status: 58, drinking status: 75, exercise status: 109, ADL score: 121, physical performance score: 224, MMSE score: 2,423, food diversity score: 526, social activity score: 26, chronic disease score: 1,291.

The risk of all-cause mortality in participants with sensory impairment were shown in Table 2. Consistent results were observed from model 1, model 2 to model 3 which fully adjusted for the potential confounders. Compared with participants without sensory impairment, participants with VI, HI or DSI had a 20% (HR=1.20, 95%CI: 1.15-1.24), 26% (HR=1.26, 95% CI: 1.21-1.31) and 46% (HR=1.46, 95% CI: 1.41–1.52) increased risk of all-cause mortality, respectively. Similar associations were observed in subgroup analyses based on gender and entry age. A significant interaction between exposure and age (*P*<0.0001 for interaction), rather than gender (*P*=0.0911 for interaction) was observed. Compared with octogenarians, nonagenarians, and centenarians, stronger association were observed among participants with an entry age less than 80 years old.

**Table 2:** Association between sensory impairment and risk of all-cause mortality among the elderly adults, a cohort study in China, 1998-2019.

**Population**	**Groups**	**Cases/Person-years**	**HR (95% CIs)**			**P for interaction**
			**Model 1**	**Model 2**	**Model 3**	
Whole Population	No sensory impairment	9128/96072	1.00 (reference)	1.00 (reference)	1.00 (reference)	
	VI only	4569/27674	1.27 (1.22-1.31)	1.27 (1.22-1.32)	1.20 (1.15-1.24)	
	HI only	4678/19323	1.38 (1.33-1.43)	1.34 (1.29-1.39)	1.26 (1.21-1.31)	
	DSI	9478/26794	1.77 (1.71-1.82)	1.73 (1.68-1.79)	1.46 (1.41-1.52)	
Male	No sensory impairment	4759/48587	1.00 (reference)	1.00 (reference)	1.00 (reference)	0.0911
	VI only	1724/10520	1.27 (1.20-1.34)	1.26 (1.19-1.34)	1.19 (1.12-1.26)	
	HI only	2014/8297	1.39 (1.31-1.46)	1.34 (1.27-1.41)	1.26 (1.19-1.33)	
	DSI	2630/7567	1.73 (1.65-1.83)	1.68 (1.60-1.77)	1.44 (1.36-1.52)	
Female	No sensory impairment	4369/47485	1.00 (reference)	1.00 (reference)	1.00 (reference)	
	VI only	2845/17154	1.27 (1.21-1.33)	1.28 (1.22-1.35)	1.21 (1.15-1.27)	
	HI only	2664/11026	1.38 (1.31-1.45)	1.34 (1.27-1.41)	1.26 (1.20-1.33)	
	DSI	6848/19227	1.78 (1.70-1.85)	1.75 (1.68-1.83)	1.48 (1.41-1.55)	
Age at baseline, <80 years	No sensory impairment	1918/50054	1.00 (reference)	1.00 (reference)	1.00 (reference)	<0.0001
	VI only	607/10145	1.36 (1.24-1.49)	1.34 (1.22-1.47)	1.29 (1.17-1.42)	
	HI only	219/2415	1.69 (1.46-1.94)	1.61 (1.40-1.86)	1.53 (1.32-1.77)	
	DSI	302/2188	2.49 (2.19-2.82)	2.36 (2.08-2.68)	2.03 (1.78-2.31)	
Age at baseline, 80-89 years	No sensory impairment	3453/29236	1.00 (reference)	1.00 (reference)	1.00 (reference)	
	VI only	1303/8543	1.20 (1.12-1.27)	1.22 (1.14-1.30)	1.15 (1.07-1.22)	
	HI only	1231/6460	1.45 (1.36-1.55)	1.40 (1.31-1.49)	1.30 (1.22-1.39)	
	DSI	1623/6538	1.77 (1.67-1.89)	1.75 (1.65-1.86)	1.50 (1.41-1.60)	
Age at baseline, 90-99 years	No sensory impairment	2520/12358	1.00 (reference)	1.00 (reference)	1.00 (reference)	
	VI only	1477/5725	1.25 (1.17-1.33)	1.25 (1.17-1.34)	1.15 (1.08-1.23)	
	HI only	1795/6372	1.34 (1.26-1.42)	1.29 (1.22-1.37)	1.18 (1.11-1.26)	
	DSI	3225/8666	1.74 (1.65-1.83)	1.69 (1.61-1.79)	1.39 (1.32-1.48)	
Age at baseline, ≥100 years	No sensory impairment	1237/4424	1.00 (reference)	1.00 (reference)	1.00 (reference)	
	VI only	1182/3260	1.30 (1.20-1.41)	1.29 (1.19-1.40)	1.18 (1.09-1.28)	
	HI only	1433/4075	1.26 (1.17-1.36)	1.23 (1.14-1.33)	1.16 (1.07-1.26)	
	DSI	4328/9402	1.66 (1.56-1.77)	1.62 (1.52-1.72)	1.35 (1.26-1.45)	

Abbreviations: CIs: confident intervals; DSI: dual sensory impairment; HI: hearing impairment; HR: hazard ratio; VI: vision impairment.

Model 1: adjusted for age and sex; Model 2: adjusted for age, sex, enrollment year, province, residence, ethic, marriage status, occupation, access to medical service, smoking status, drinking status, and exercise status; Model 3: model 2 + further adjusted for ADL score, physical performance score, MMSE score, food diversity score, social activity score, and chronic disease score.

In sensitivity analysis, we observed participants with hearing aids had an increased risk of all-cause mortality (HR=1.06, 95% CI: 1.03-1.09) (Supplementary Table 1). We also assessed the risk of all-cause mortality among participants with newly incident sensory impairment (Table 3) and observed similar but stronger associations. The risk of all-cause mortality increased 22% (HR=1.22, 95% CI: 1.14-1.29), 39% (HR=1.39, 95% CI: 1.29-1.49) and 80% (HR=1.80, 95% CI: 1.80-1.91) among participants with VI, HI or DSI, respectively.

**Table 3:** Risk of all-cause mortality among the elderly adults with newly incident sensory impairment, a cohort study in China, 1998-2019.

**Population**	**Groups**	**Cases/Person-years**	**HR (95% CI)**		
			**Model 1**	**Model 2**	**Model 3**
Whole Population	No sensory impairment	3405/39342	1.00 (reference)	1.00 (reference)	1.00 (reference)
	VI only	1411/11323	1.22 (1.15-1.30)	1.23 (1.16-1.31)	1.22 (1.14-1.29)
	HI only	1041/4947	1.47 (1.37-1.58)	1.41 (1.32-1.52)	1.39 (1.29-1.49)
	DSI	1668/5624	1.88 (1.76-2.00)	1.87 (1.75-1.99)	1.80 (1.69-1.91)

Abbreviations: CIs: confident intervals; DSI: dual sensory impairment; HI: hearing impairment; HR: hazard ratio; VI: vision impairment.

Model 1: adjusted for age and sex; Model 2: adjusted for age, sex, enrollment year, province, residence, ethic, marriage status, occupation, access to medical service, smoking status, drinking status, and exercise status; Model 3: model 2 + further adjusted for ADL score, physical performance score, MMSE score, food diversity score, social activity score, and chronic disease score.

## DISCUSSION

In this large population-based cohort study, we found that sensory impairment was associated with higher risk of all-cause mortality among the elderly population in China. Compared with participants without sensory impairment, those with VI, HI and DSI had a 20%, 26% and 46% increased risk of all-cause mortality, respectively. The associations were robust in subgroup analyses and sensitivity analyses.

Our finding was consistent with results from previous studies [5, 16–18]. A six-year follow-up study in Italy showed that hearing deficit was associated with a significant increased mortality risk [17]. One study of 1754 adults aged 65 or older from Japan reported increased risk of mortality in those with DSI and cognitive impairment [5]. A large European study found that DSI was associated with increased mortality at nursing homes [18], and similar result was also observed in Australian older adults [16]. However, there are some studies failed to observe positive association as well [6, 13, 14, 19]. For example, one study conducted in Japan found the association between VI and mortality was attenuated and became non-significant after adjustment (HR 1.05; 95% CI, 0.83–1.32) [13]. Among the 1658 participants from National Health and Nutrition Examination Survey, HI and VI were not independently associated with all-cause mortality [14], and non-significant result was also observed in Epidemiology of Hearing Loss Study participants [19]. The conflicting findings are probably due to the heterogeneity in studied ethnicity, age distribution of enrolled population, study design, sample size, or definition criteria of VI or HI. Some studies objectively measured sensory impairment by using medical tools such as sound-treated booth or Early Treatment Diabetic Retinopathy Study charts [19], while others used subjective assessment by interviewers [5, 6] or self-report [14]. One study that used data from CLHLS was also explored the association between sensory impairment and all-cause mortality [6]. However, compared to our results, much lower effect estimates and wider 95% CIs were observed, which might be caused by smaller sample size, shorter follow-up time, and misclassification of exposure status (without considering the change of sensory impairment after enrollment).

We observed the effects of sensory impairment on mortality risk decreased with aging. Similar deceasing pattern was also found in other well-established mortality risk factors among younger adults, such as increased body mass index, hypertension, hypercholesterolemia, cancer and heart disease, which may not continue to pose a risk to the oldest-old adults [20]. Such deceasing pattern may be explained by the increased number of chronic diseases and function inabilities related to mortality risk with increasing age, which makes the effect of any single disease or inability on mortality less important [20].

The precise mechanisms regarding to the association between sensory impairment and all-cause mortality remain unclear. Cognitive impairment, reported to be associated with higher mortality [21], may be one of the underlying mechanisms. Sensory impairment may be associated with cognitive impairment through age-related neurological changes (e.g., neurodegeneration in the central nervous system, or neuronal atrophy due to decreased sensory input [22]), and vascular changes (e.g., atherosclerotic or microvascular changes) [23, 24]. A causal relationship between them was also suggested through increased cognitive load, sensory deprivation, depression, or social isolation [25]. Besides, the increased risk of all-cause mortality observed in persons with sensory impairment might be mediated by factors known to increase the risk of sensory impairment in the elderly adults (e.g., hypertension, diabetes, accident and injury) [16]. Furthermore, sensory impairment may be a proxy for frailty (e.g., handgrip strength, peak expiratory flow), which is also a strong predictor of mortality [26].

Our study strengthens and extends previous findings in three aspects. First, the present study based on a large population-based cohort with verified outcomes and abundant covariate information, which enable us to do informative subgroup analyses and sensitivity analyses. Second, taking into account the variation of sensory impairment status during the follow-up period could avoid immortal time bias and more precisely assess the association. Moreover, this nationwide representative sample of Chinese was mainly consisted of the oldest-old population, such as octogenarians, nonagenarians, and centenarians, where the influence of sensory impairment on all-cause mortality was rarely explored. As aging is an inevitable trend, it is critical to promote the health of the elderly through the study on the longevity [3].

However, several limitations of our study should not be ignored. First, misclassification of exposure status could be a concern. Although all interviewers were required to attend a series of standardized training sessions before commencing interviews, subjective assessment of sensory impairment could underestimate the real prevalence in comparison with objective assessment, as suggested in one study [27]. Besides, background noise and voice variations of interviewers during hearing function assessment could further misclassify exposure status. However, as such misclassifications are unlikely to be differential, they are likely to dilute the real association towards the null. Second, as cognitive impairment affects a person’s ability of correctly understanding, following instructions or interpreting communication [28]. Subjective evaluation precludes the possibility of distinguishing participants with sensory impairment between those with cognitive impairment or those with combined impairments in sensory and cognitive. Third, unlike randomized trails, our study is prone to residual confounding due to unmeasured or imprecisely measured confounders. However, the robust results observed in subgroup analyses and sensitivity analyses argue against that residual confounding is a big concern here. Fourth, as many of the participants die at home and cause of death of deceased participants was reported by family number, the association between sensory impairment and cause-specific mortality was unable to be investigated. Fifth, lacking of information on the reason of sensory impairment makes us unable to further explore whether the influence of sensory impairment on all-cause mortality differs among population with different underlying conditions that caused sensory impairment, such as congenital and acquired. Finally, because the majority of studied population was the oldest-old Chinese and subjective assessment leads to underestimation of sensory impairment among them, the generalization of our findings to other areas, ethnicities and assessment methods should be considered with caution.

In summary, our findings demonstrated that sensory impairment were associated with higher risk of mortality among the elderly adults in China, which indicates sensory impairment might be a signal for identifying the individuals at a higher risk of death. Healthcare providers therefore should be aware of the increased risk of mortality in those with sensory impairment in their routine clinical practices.

## MATERIALS AND METHODS

### Study design

We performed a cohort study by using data from the Chinese Longitudinal Healthy Longevity Survey (CLHLS). Detailed information of CLHLS has been previously published [29]. Briefly, CLHLS, initiated in 1998, is a nationwide survey conducted in a randomly selected half of the counties and cities in 22 of the 31 provinces, covering about 85 percent of the total population [4]. A total of 56,949 participants that enrolled in its eight waves, conducted in 1998, 2000, 2002, 2005, 2008-2009, 2011-2012, 2014 and 2018-2019, respectively, were enrolled and have been followed up since enrollment. In present study, we excluded 19,226 participants with only baseline information (due to newly enrolled in 2018-2019 wave or immediately lost to follow-up after baseline survey) and 647 participants without information on sensory status, birthday or end of follow up time. Eventually, a total of 37,076 participants were included in the main analyses. A total of 11,365 participants without sensory impairment at baseline and having at least one more follow-up record were further enrolled in the sensitivity analysis of exploring the association of sensory impairment and risk of all-cause mortality. The flow chart of participant selection was shown in the Supplementary Figure 1.

Research Ethics Committees of Peking University approved the CLHLS, and consent forms from all participants or their representatives were also collected before participation.

### Sensory impairment and death assessment

Vision function was assessed by questions of asking participant whether he/she, after taking off correction (e.g., glasses), could see the circle on a card and distinguish the direction of the break in the circle. The circle was positioned in one meter away from all participants. There are four choices: a). can see the circle and distinguish the direction of the break; b). can see the circle but unable to distinguish the direction of the break; c). cannot see; d). blind. Participants that unable to distinguish the direction of the break or worse were categorized as having vision impairment.

Hearing function was assessed by the interviewers if the participant can clearly hear what the interviewer asked during the interview. There are four choices: a). yes, without hearing aid; b). yes, but with hearing aid; c). can partially hear, with hearing aid; d). cannot hear. Participants that needing hearing aid during interview or worse hearing condition were categorized as having hearing impairment.

Based on results of the two questions, we then categorized the whole population into four groups: a). no sensory impairment; b). vision impairment (VI); c). hearing impairment (HI); and d). dual sensory impairment (DSI).

Date of death was acquired and confirmed by family member or the village doctor. Risk of all-cause mortality was the interested outcome. Cause-specific mortality, however, was not considered in present study due to two main reasons: a). many of the elderly individuals die in a natural way at home rather than in hospital where cause of death may be recorded; b). cause of death of deceased participants reported by family number were imprecise and unreliable [30].

### Covariates assessment

Sociodemographic information, lifestyle factors and health status of participant was considered in present study to minimize the effect of potential confounders. Sociodemographic information included age, sex (male/female), enrollment year (categorical variable: 1998, 2000, 2002, 2004, 2008, 2011, and 2014), province (categorical), residence (city, town and rural area), ethic (Han/others), marriage status (married/others (e.g., widowed, divorced or never married)), occupation (farmer or manual, clerical, professional, and others) and access to medical service (yes/no). Lifestyle factors included smoking (never, ever, and current smoker), drinking (never, ever, and current drinker), exercise (never, ever, and current exerciser), food diversity intake (measured by food diversity score), and social activity engagement (measured by social activity score). Health status information included disability of activities in daily living (ADL, measured by ADL score), disability of physical performance (measured by physical performance score), cognitive impairment (measured by Mini-Mental State Examination (MMSE) score) and chronic disease status (measured by chronic disease score). The definition of the abovementioned scores was described in the Supplementary Table 2.

### Statistical analyses

Cox regression models with age as the underlying time scale were used to explore the association between sensory impairment and all-cause mortality. The proportional hazard assumption was evaluated via Schoenfeld residual plots and no violation was observed. All participants were followed from enrollment until death, lost to follow-up, or 31 July 2019, whichever came first. Because the sensory status could change after enrollment, failing to consider this change would introduce misclassification of the exposure status and lead to immoral time bias [31]. We therefore used sensory status as a time-varying exposure. For example, a participant contributed person-years to “no sensory impairment” since he/she was free of sensory impairment, and then contributed person-years to the “HI” from the date of being recorded with HI, until he/she was recorded with a different another record or he/she reached one of the follow-up end points.

To test the robustness of the findings, we performed three models to adjust for potential confounders. In model 1, we included age (underlying time scale) and sex as covariates. In model 2, we further adjusted for enrollment year, province, residence, ethic, marriage status, occupation, access to medical service, smoking, drinking and exercise. In model 3, the covariates in model 2 and the following covariates were also adjusted for: activities in daily living score (categorical: 6, 5, 3-4, and 0-2), physical performance score (categorical: 5, 2.5-4.5, and 0-2.5), MMSE score (categorical: 24-30, 18-23, and 0-17), food diversity score (categorical: 6-8, 4-5, and 0-3), social activity score (categorical: 5-8, 3-4, and 0-2), and chronic disease score (categorical: 0, 1-2, and ≥3). Missing information on covariates were replaced by using a missing indication.

We also performed subgroup analyses according to the baseline information: baseline age and sex. Testing for heterogeneity between age groups or sex was performed by including an interaction term of the exposure and age or sex in the Cox regression model. We conducted a sensitivity analysis among those with hearing aids (n=9,389), as those might be a slightly better-defined category of hearing loss. Given the risk of all-cause mortality among prevalent cases, that is participants with sensory impairment before enrollment, may be different from the incident cases due to changing lifestyle factors after having sensory impairment, we, as a sensitivity analysis, further excluded individuals with sensory impairment at baseline and repeated main analysis among incident cases.

Data analyses were performed using SAS version 9.4 (SAS Institute Inc, Cary, NC). A two-sided *P≤* 0.05 was considered statistically significant for the primary exposure.

### Availability of data and materials

All data used in this study was stored at Peking university (http://opendata.pku.edu.cn/) and available upon request.

## Supplementary Material

Supplementary Figure 1

Supplementary Tables
