# Supplementary material for: Sensory impairment and all-cause mortality among the elderly adults in China: a population-based cohort study
Source: Aging (Albany NY). 2020 Nov 26;12(23):24288–300. doi: 10.18632/aging.202198 (PMC7762477; doi:10.18632/aging.202198)
Supplement: Supplementary Figure 1 [file aging-12-202198-s001.pdf]

SUPPLEMENTARY FIGURE

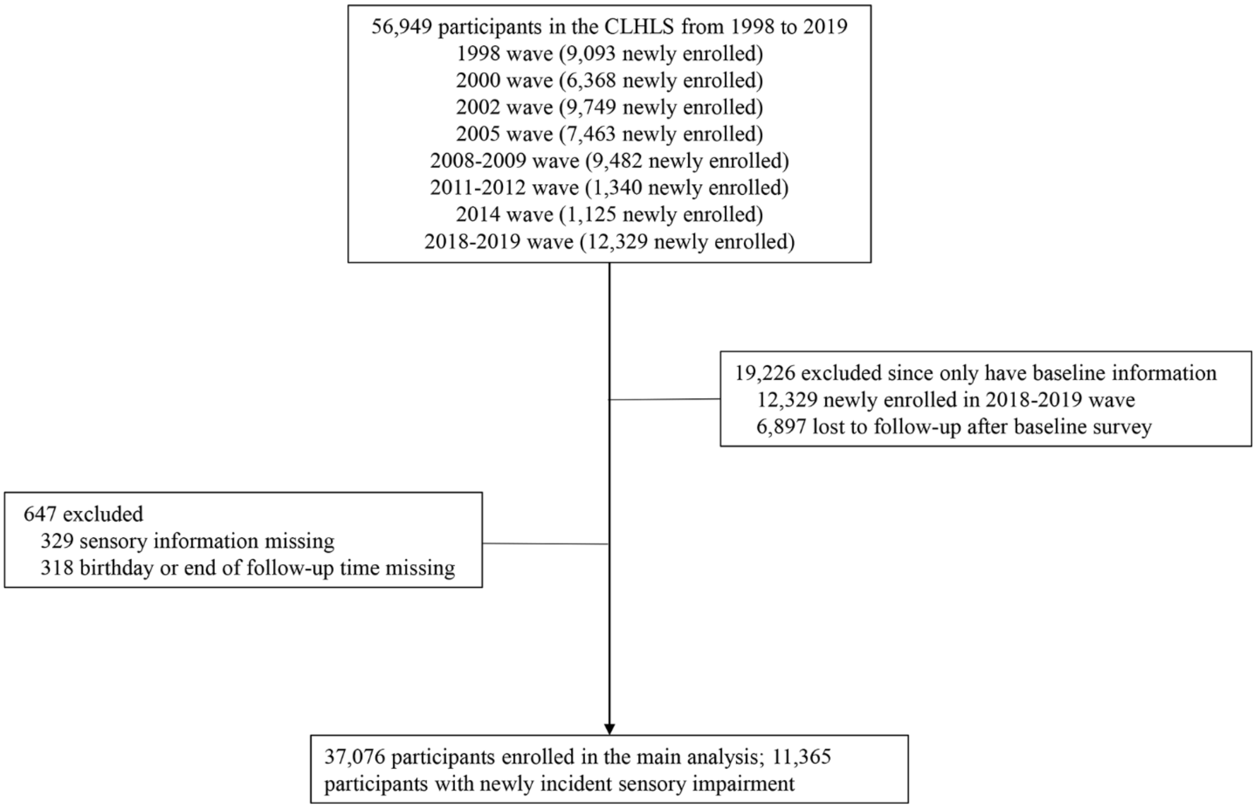

Supplementary Figure 1. Flow chart of participant selection, a cohort study of the elderly adults in China, 1998-2019.
