# Supplementary material for: Sensory impairment and all-cause mortality among the elderly adults in China: a population-based cohort study
Source: Aging (Albany NY). 2020 Nov 26;12(23):24288–300. doi: 10.18632/aging.202198 (PMC7762477; doi:10.18632/aging.202198)
Supplement: Supplementary Tables [file aging-12-202198-s002.pdf]

SUPPLEMENTARY TABLE

Supplementary Table 1. Risk of all-cause mortality among participants with hearing aid, a cohort study in China, 1998-2019.

Population	Groups	Cases/Person-years	HR (95% CIs)		
			Model 1	Model 2	Model 3
Whole Population	No sensory impairment	16072/132449	1.00 (reference)	1.00 (reference)	1.00 (reference)
	Participants with hearing aid	8343/29047	1.26 (1.23-1.30)	1.23 (1.19-1.26)	1.06 (1.03-1.09)

Abbreviations: CIs: confident intervals; HR: hazard ratio.
Model 1: adjusted for age and sex; Model 2: adjusted for age, sex, enrollment year, province, residence, ethic, marriage status, occupation, access to medical service, smoking status, drinking status, and exercise status; Model 3: model 2 + further adjusted for ADL score, physical performance score, MMSE score, food diversity score, social activity score, and chronic disease score.

Supplementary Table 2. Definition of scores that analyzed in this article.

Score	Measured for	Items	Assignment	Range
Activities of daily living (ADL) score	Disability of ADL	Six items regarding ADL: eating, dressing, transferring, using the toilet, bathing and continence	If there was no need assistance for the activity, one score was given, otherwise zero was given.	Ranging from 0 to 6; Higher score indicating better functional capacity
Mini-Mental State Examination (MMSE) score	Cognitive impairment	Twenty-four items regarding orientation, registration, attention, calculation, recall and language	For 23 items, one score was given if the participants rightly answered the question; for the last item, zero to 7 score was given based on the number of the name of eatable foods that participants mentioned within 1 minute.	Ranging from 0 to 30; Higher score indicating better cognitive function
Food diversity score	Diversity of food intake	Consumption frequency of eight food groups: fruits, vegetables, meat, fish, eggs, legumes and its products, garlic, tea	If the consumption frequency of one food group was at least once a week, one score was given, otherwise zero was given.	Ranging from 0 to 8; Higher score indicating higher food diversity
Social activity score	Social activity engagement	Taking frequency of eight social activities: doing outdoor activities, raise pets, reading books, watching TV or listening to the radio, doing housework or taking care of grandchildren, playing cards or mah-jong, taking part in social activities or religious activities	If the taking frequency of one activity was at least once a week, one score was given, otherwise zero was given.	Ranging from 0 to 8; Higher score indicating higher social activity engagement
Physical performance score	Disability of physical performance	Five objective physical performance tests: standing from a chair, picking up a book from the floor, being able to turning around 360 degree, being able to touch neck by hand and being able to touch lower back by hand	If the participant can finish one test without help or by using two hands then one score was given; if they can finish it with help or only using one hand then “0.5” was given; otherwise zero was given.	Ranging from 0 to 5; Higher score indicating better physical capacity
Chronic disease score	Chronic disease status	Status of eight chronic disease: hypertension, diabetes, heart disease, stroke or cerebrovascular disease, lung disease (e.g., bronchitis, emphysema, asthma or pneumonia), cancer, gastrointestinal ulcer and Parkinson’s disease	If the participant self-reported or was diagnosed with the specific disease then one score was given, otherwise zero was given.	Ranging from 0 to 8; Higher score indicating higher chronic disease status
